# U. S. Stamp Duties and Taxes

**Published:** 1862-11

**Authors:** 


U. S. STAMP DUTIES AND TAXES.—Messrs. T. B. Peterson & Brothers, 306 Chestnut Street, Philadelphia, have just issued a neat card, containing a list of "Stamp Duties" imposed by the Act of 1862, which Act went into effect on the 1st of October. The card will be found very convenient for reference by all, and should be at the side of every storekeeper, merchant, manufacturer, broker, banker, attorney, or any man of business, as it shows at a glance the amount of stamp duty or tax to be paid on everything in every-day business, as well as the penalties of the law, and fines for trying to evade each and every one of the Stamp Taxes imposed by Congress. It will save a world of trouble to every storekeeper and business man to have a copy for reference at their side. It has been carefully prepared from the Official Documents at Washington, and copyrighted by a noted member of the Philadelphia Bar. Copies will be sent per mail everywhere by the publishers, on receipt of the price.

